# Application of clinical indicators in evaluating vestibular compensation efficacy in benign recurrent vestibular vertigo patients with short-term personalized vestibular rehabilitation

**DOI:** 10.1007/s00405-024-08457-8

**Published:** 2024-01-23

**Authors:** Jinyu Wang, Yibo Lei, Liang Tian, Jinjing Zuo, Yayun Shen, Jing Wang

**Affiliations:** 1 Ear, Nose, Throat (ENT) Institute and Department of Otorhinolaryngology, Eye and ENT Hospital, Fudan University, Shanghai, 200031 China; 2 Shanghai Auditory Medical Center, Shanghai, 200031 China; 3 NHC Key Laboratory of Hearing Medicine, Fudan University, Shanghai, 200031 China; 4 Department of Otology, Otolaryngology Hospital, The First Affiliated Hospital, Zhengzhou University, Zhengzhou, 450052 Henan China

**Keywords:** Benign recurrent vestibular vertigo, Vestibular rehabilitation, Short-term personalized program, Efficacy grading

## Abstract

**Background:**

Short-term personalized vestibular rehabilitation (ST-PVR) can establish stable vestibular compensation. However, there is a lack of a clear definition for clinical indicators that can dynamically reflect the progress of vestibular rehabilitation (VR).

**Objective:**

To explore the clinical indicators suitable for evaluating the effectiveness of ST-PVR in treating benign recurrent vertigo (BRV).

**Methods:**

In total, 50 patients diagnosed with BRV were enrolled. All patients received the ST-PVR treatment program. At 2 and 4 weeks after rehabilitation, subjective scales, including the visual analogue scale (VAS), dizziness handicap inventory scale (DHI), activities‐specific balance confidence scale (ABC) and generalized anxiety disorder (GAD-7) were assessed. Objective vestibular function tests were performed. VR grading was determined.

**Results:**

At 2 weeks after rehabilitation, significant enhancements were observed in VAS, DHI, ABC, GAD-7, UW, vHIT results, and VR grading scores (*p* < 0.05). The sensory organization test (SOT) results demonstrated statistically significant improvements at 2 weeks and 4 weeks after rehabilitation (*p* < 0.05).

**Conclusion and significance:**

Both subjective scales and partial examination results in objective assessment can serve as indicators to dynamically monitor the compensatory process of vestibular function in patients with BRV. The VR efficacy grading score, which incorporates the above indicators, allows for quantification of the changes that occur during the vestibular rehabilitation process.

## Introduction

Vertigo refers to the feeling of self-movement in the absence of self-movement, is a prevalent condition in clinical practice and spans across multiple disciplines [1]. Large population-based studies revealed that vertigo affects approximately 15% to over 20% of adults on annual basis, with a lifetime prevalence of 7.4% among adults aged 18–79 and the prevalence of vertigo is observed to increase annually at a rate ranging from 7.6 to 17% [2–4]. Vestibular vertigo is a common subtype of vertigo with a 12-month prevalence rate of 5% and an annual incidence of 1.4%. Notably, its prevalence tends to increase with age, and women are approximately two to three times more susceptible to this condition than men [5, 6]. Vestibular vertigo can significantly impact the daily life of individuals, hinder mental functioning and cause psychological disorders such as anxiety and depression [7]. Research investigated that the rate of missed work owing to vestibular vertigo is about 40% and approximately 18.5% of individuals encounter difficulties in performing their regular activities [8, 9].

Benign recurrent vertigo (BRV) is a term used to describe a group of peripheral vestibular symptoms without neurological or cochlear abnormalities, patients experience recurrent episodes of vertigo lasting from several minutes to days, with varying frequencies ranging from daily to every few years. Importantly, BRV does not meet the diagnostic criteria for other vestibular disorders [10]. BRV was first described by Slater in 1979 [11], and a unified consensus on its concept is still lacking. Hitherto, the pathogenesis and etiology of BRV still remain unclear. Several studies suggest that BRV is highly associated with migraine or vestibular migraine (VM) with high comorbidity between the above diseases, while others claim the involvement of Ménière disease (MD) [12–15]. Recurrent episodes of vertigo can be triggered by attacks of underlying primary diseases. However, vestibular decompensation, which refers to the absence of established vestibular compensation or the reoccurrence of previously established compensation, can lead to recurrent vertigo attacks [16, 17]. Vestibular rehabilitation has been recognized as an effective treatment approach for these patients [17]. Vestibular rehabilitation is an exercise-based program that focuses on enhancing the function of the vestibulo-ocular reflex (VOR) to maintain visual stability [18], improving the function of the vestibulo-spinal reflex (VSR) to enhance balance during daily activities [19], and increasing tolerance to head and body movements to reduce motion sensitivities [20]. The purpose of VR is to establish stable vestibular compensation, improve the patient's vestibular function and enhance their balance ability [21].

Based on the vestibular rehabilitation clinical practice guideline for patients with peripheral vestibular hypofunction in 2016 and the updated version in 2022 [22, 23], the effectiveness of vestibular rehabilitation in improving the symptoms and functions of patients with unilateral and bilateral vestibular hypofunction has been strongly evidenced [24]. The concept of short-term personalized (ST-PVR) has emerged as a recent approach in the field of vestibular rehabilitation. The key features of ST-PVR include its short duration, personalized approach, and intensive rehabilitation sessions. It has been suggested that ST-PVR can lead to rapid and effective promotion of the stable establishment of vestibular compensation while addressing the specific needs of each patient for better treatment outcomes and offering significant advantages in the treatment of decompensated vestibular vertigo patients [17, 25].

Based on a comprehensive evaluation of the patient’s vestibular function, ST-PVR was developed and continually adjusted to accommodate changes in different clinical stages. This emphasizes the importance of shortening the training time while ensuring therapeutic effectiveness. This approach aims to reduce training stress while maintaining exercise accuracy, improving treatment efficiency, and ultimately reducing or eliminating symptoms and enhancing the patient's quality of life. In our previous study, we found that ST-PVR significantly increased the patient's treatment compliance due to the shorter total time needed for daily training [17].

The lack of a clear definition for clinical indicators that can dynamically reflect the progress of vestibular rehabilitation and indicate the establishment of vestibular compensation is a challenge in the field. In this study, we observed and defined clinical indicators that exhibit dynamic changes during ST-PVR. Additionally, we assessed the feasibility of formulating a grading standard for evaluating the efficacy of vestibular rehabilitation by comprehensively analyzing these clinical indicators.

## Materials and methods

### Clinical data

In this prospective observational study, a total of 50 patients (16 men and 34 women, aged 18–79) diagnosed with benign recurrent vestibular vertigo who visited Eye and Ear, Nose and Throat (EENT) Hospital of Fudan University from October 2021 to March 2022 were enrolled. The inclusion criteria for the study were as follows: (1) age between 18 and 80 years; (2) experiencing benign recurrent vertigo in the past month, characterized by transient episodes of vertigo lasting for several seconds to minutes on most days, or vertigo lasting more than ten minutes to days, occurring at least three times or more; (3) presence of symptoms such as dizziness, imbalance, or visual disturbances between episodes; and (4) absence of other neurological symptoms in the past month, such as changes in hearing. The exclusion criteria for the study included patients diagnosed with any central structural diseases, migraine, other peripheral vertigo disorders such as benign paroxysmal positional vertigo (BPPV), VM, MD, or sudden sensorineural hearing loss accompanied by vertigo. Patients who were unable to cooperate with the ST-PVR protocol were also excluded.

Ethical approval for the study was obtained from the medical ethics committee of the EENT Hospital of Fudan University (approval number: 2017043), and all participants provided written informed consent prior to their inclusion in the study.

### Baseline assessment and follow-up

#### Demographic data

The demographic information collected from each participant included gender, age, the duration of symptoms, and the average monthly episodes of vertigo. These parameters provide a basic understanding of the study population.

#### Physical examination

Physical examination includes the assessment of vertigo and balance disorder symptoms, which involved the following tests: (1) Spontaneous Nystagmus (Nys) was examined to assess its presence, direction, and intensity, aiming to identify abnormalities in the vestibular system [26]. (2) The Romberg Test (Rom) evaluated individuals' static balance maintenance, where any swaying or loss of balance indicated potential impairments in proprioception or vestibular function. [27]. (3) The Head Thrust Test (HTT) assessed the integrity of the VOR. If the patient's eyes are unable to maintain fixation on the target, it suggests a dysfunction in the VOR [28].

#### Subject scales

Subject scales were used to assess various aspects related to vertigo, balance, handicap, confidence, and anxiety [29], and the scales employed were as follows:

1. Visual Analogue Scale (VAS): The VAS is a self-report scale used to measure the intensity or severity of a particular symptom. In the context of this study, the VAS was used to assess the subjective perception of vertigo or dizziness intensity [29]. Participants are typically asked to rate their symptoms on a horizontal line, with the endpoints representing extreme severity (e.g., "no dizziness" to "worst dizziness imaginable").
2. Dizziness Handicap Inventory Scale (DHI): The DHI is a validated questionnaire that assesses the impact of dizziness on daily life. It includes items related to physical, functional, and emotional aspects affected by dizziness [30]. Scores are summed to measure dizziness-related handicap or disability, with a higher score indicating a greater impact on daily activities and quality of life [31].
3. Activities-Specific Balance Confidence Scale (ABC): The ABC scale measures self-perceived confidence in performing activities without losing balance or experiencing dizziness. Participants rate their confidence level from 0% (no confidence) to 100% (complete confidence) for each activity listed. The scores are summed for an overall measure of balance confidence [32].
4. Generalized Anxiety Disorder Scale (GAD-7): The GAD-7 is a self-report questionnaire for assessing the severity of generalized anxiety disorder symptoms. It includes seven items that capture common anxiety symptoms experienced over the past two weeks. Participants rated the frequency of each symptom. The scores are summed to measure of anxiety severity, with higher scores indicating greater anxiety symptomatology [33].

#### Objective assessment of vestibular function

1. Sensory Organization Test (SOT) using Computerized Dynamic Posturography [CDP, (EquiTest System, Neurocom Inc., USA)]: The SOT is a test that evaluates a person's ability to maintain balance under different sensory conditions. The participant stands on a force platform and their postural sway is measured. The test assesses the integration of visual, vestibular, and somatosensory inputs for balance control [10].
2. Caloric Test (CT) using Video-Electronystagmography (VNG) Test: The caloric test is performed using VNG (Otometrics, Taarstrup, Denmark), which records eye movements via video goggles. Warm or cool air or water is introduced into each ear canal to stimulate the vestibular system and the resulting nystagmus was recorded and analyzed to assess the vestibular function. Unilateral weakness (UW) and directional preponderance (DP) are parameters used to quantify vestibular asymmetry, and UW > 22% and/or DP > 25% were considered abnormal [34].
3. Video-Head Impulse Test (vHIT): The vHIT (ICS Impulse, GN Otometrics, Taastrup, Denmark) is a diagnostic test that evaluates the function of the VOR. It measures the eye movement response to rapid head movements using high-speed video goggles or cameras. The participant focuses on a target while the examiner delivers quick and unpredictable head impulses in different directions [35, 36].

In addition to the baseline evaluation, this study included two follow-up visits at 2 weeks and 4 weeks after the treatment. During the follow-up visits, various assessments, including subjective and objective measures, might have been performed to track changes in symptoms, functional outcomes, and vestibular function.

### Clinical evaluation indicators and grading standard (Table 1)

**Table 1:** Vestibular rehabilitation efficacy grading standard from Chinese Experts Consensus in Vestibular Rehabilitation [37]

Assessment indicators	3 points	2 points	1 point	0 points
Physical Examination				
Nys	Normal	Fixation instability	Grade 1	Grade 2–3
Rom	Normal	Grade1: Mild, slight sway)	Grade 2: Moderate, wide sway	Grade3: Severe, falls
HTT	Normal	Fixation instability	Microscopic saccade	Overt saccade
Subjective Scales				
VAS (scores)	0	1–3	4–6	7–10
DHI (scores)	0	≤ 30	31–60	61–100
ABC (%)	81–100	67–80	31–66	0–30
Objective Tests				
CDP/SOT	Normal	Balance total scores within 15 points below the normal range	Below the normal range with a difference greater than 15 points, no falls	Falls
VNG/CT	Normal	Abnormal DP value	Abnormal DP value with positional Nys	Abnormal DP value with spontaneous Nys
vHIT	Normal	Gain reduction with covert saccade	Gain reduction with covert and overt saccade	Gain reduction with overt saccade

Nys: nystagmus test; Rom: Romberg test. HTT: head thrust test; VAS: visual analogue scale; DHI: dizziness handicap inventory; ABC: activities‐specific balance confidence scale; CDP: computerized dynamic posturography; SOT: sensory organization test; VNG: video‐electronystagmography; CT: caloric test; vHIT: video-head impulse test; DP: directional preponderance

To facilitate the prompt assessment of patients' vestibular recovery, a graded scoring standard can be established to evaluate the effectiveness of vestibular rehabilitation. The selection of the above nine clinical indicators for physical examination, subjective scales, and vestibular function tests were based on previous research results and the vestibular rehabilitation efficacy rating standard proposed in the Chinese expert’s consensus on vestibular rehabilitation [37]. These indicators offer a comprehensive assessment of various aspects related to vertigo, balance, handicap, confidence, anxiety, and vestibular function. They collectively provide insights into the status of vestibular compensation.

The following evaluation indicators are assigned scores based on the level of recovery observed: a score of 3 indicates complete restoration to a normal state, a score of 2 represents mild abnormalities, a score of 1 corresponds to moderate abnormalities, and a score of 0 indicates severe abnormalities. This scoring system enables a quick determination of the patient's vestibular rehabilitation outcome. Based on the evaluation of 9 indicators related to vestibular function, a total score of 27 indicates complete rehabilitation, a score range of 18–26 suggests substantial recovery, a score range of 9–17 indicates partial recovery and a score range of 0–8 indicates no recovery.

### Interventions

**Fig. 1:**
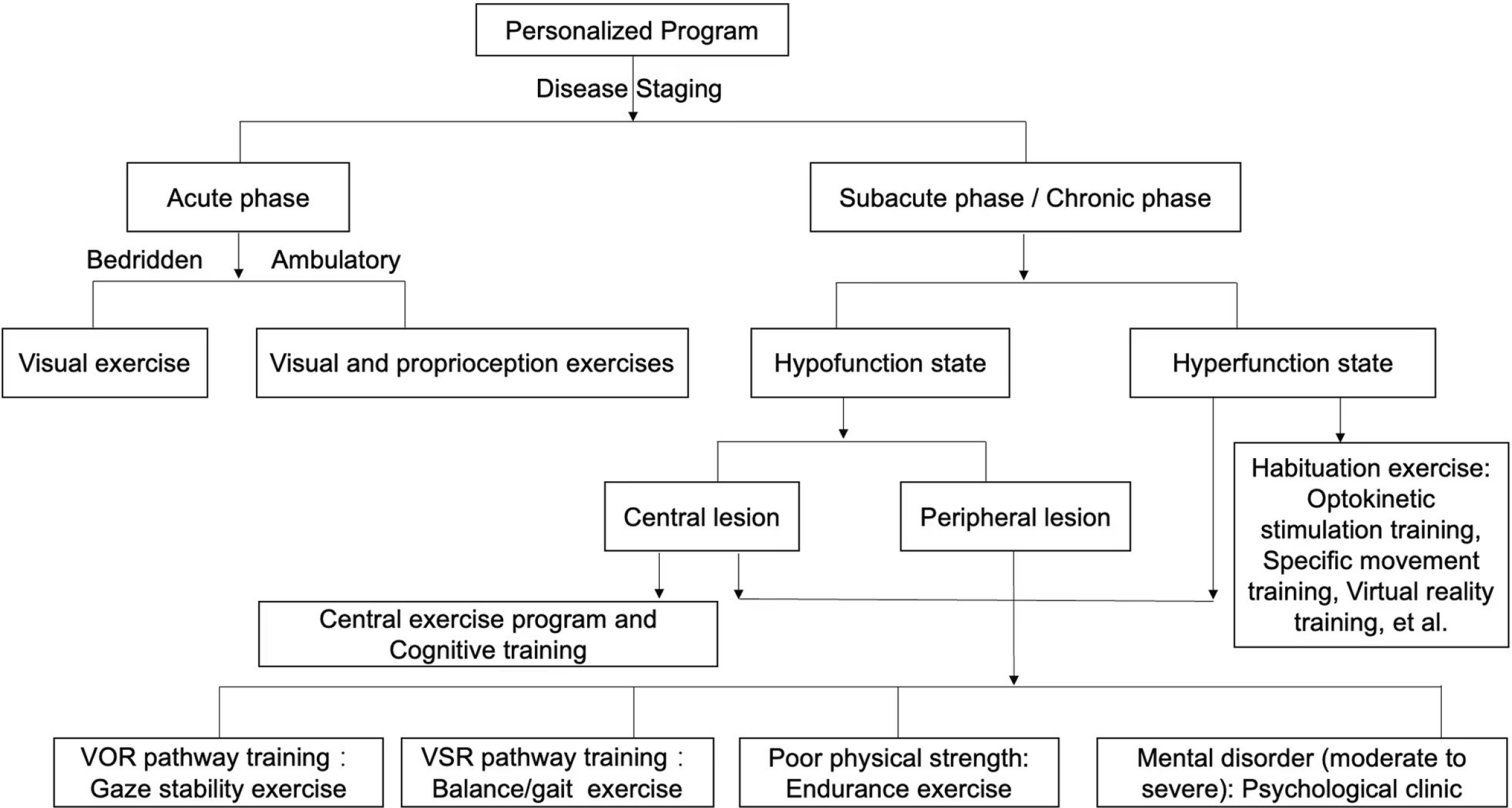
Flowchart of formulation strategy for personalized vestibular rehabilitation program [37]

The formulation strategy of ST-PVR is listed in Fig. 1 [37]. Gaze stability exercises such as "vorx1" and "vorx2" involve fixedly looking at a stationary target and a target moving in the opposite direction while performing sinusoidal head rotations. Balance exercises include maintaining balance during visual and proprioceptive changes and adjusting foot support surfaces for increased difficulty. Center of gravity transfer training improves control and balance. Gait training involves walking under various dynamic conditions. These exercises enhance coordination and mobility.

The selection of specific vestibular rehabilitation exercises should be based on factors such as the disease stage, functional status, and lesion location of the patients. In the ST-PVR program, it is recommended that patients engage in training sessions lasting 30 s each, with approximately 2–8 sessions per session and three sessions per day. As a result, patients only need to dedicate 3–12 min of training time each day.

### Statistical analysis

All statistical analyses were performed using SPSS 26.0 software (SPSS Inc., Chicago, IL, USA). Normally distributed continuous variables are presented as the mean ± standard deviation (SD). Categorical variables were compared using the chi-square test. The paired t test was used to compare differences before and after treatment for normally distributed continuous variables. For continuous or ranked data with skewed distribution, the Mann‒Whitney *U* test and the Kruskal‒Wallis *H* test were employed. A *p* value of 0.05 was considered the threshold for statistical significance.

## Results

### Basic clinical data of evaluation indexes

A total of 50 patients, including 16 males and 34 females, were enrolled in this study. The age of the participants ranged from 18 to 79 years old, with a mean age of 48 ± 11 years. Among the participants, 24 cases were aged 50 years or younger, while 26 cases were older than 50 years. The duration of vertigo symptoms and the average monthly episodes of vertigo showed considerable variation among the patients. The duration of symptoms ranged from 1 min to 2 days (mean of 5.99 ± 11.53 h), while the average monthly episodes of vertigo ranged from 1 to 20 episodes (mean of 4.20 ± 4.67 episodes). Table 2 presents the numbers and proportions of patients with abnormal results in each evaluation index at baseline and after ST-PVR treatment. The correlation between the baseline assessment results and various patients’ variables, including age, gender, duration of symptoms, and the average monthly episodes of vertigo, was analyzed using the Mann‒Whitney U test. However, no statistically significant correlation was found (*P* > 0.05).

**Table 2:** Number and proportion of patients with abnormal results in each evaluation index at baseline and after treatment

Evaluation index	Baseline (0-week, *n* = 50)				2 weeks (*n* = 50)		4 weeks (*m* = 50)	
	Age (P value)	Gender (P value)	Abnormal cases (n)	Abnormal rate	Abnormal cases (n)	Abnormal rate	Abnormal cases (n)	Abnormal rate
**Nys**	0.227	0.859						
FI			12	24%	8	16%	8	16%
Grade 1			2	4%	2	4%	0	0%
Grade 2			0	0%	0	0%	0	0%
Grade 3			2	4%	0	0%	0	0%
**Rom**	0.105	0.502						
Grade 1			8	16%	5	10%	4	8%
Grade 2			1	2%	0	0%	0	0%
Grade 3			1	2%	0	0%	0	0%
**HTT**	0.604	0.896						
Abnormal			3	6%	0	0%	0	0%
**VAS**	0.171	0.643						
Mild			19	38%	38	76%	41	82%
Moderate			18	36%	10	20%	7	14%
Severe			13	26%	2	4%	2	4%
**DHI**	0.793	0.323						
Normal			1	2%	2	4%	3	6%
Mild			10	20%	22	44%	35	70%
Moderate			26	52%	22	44%	10	20%
Severe			13	26%	4	8%	2	4%
**ABC**	0.763	0.328						
< 80			32	64%	13	26	4	8%
**SOT**	0.152	0.909						
< 70			10	20%	8	16%	4	8%
**VNG**								
DP ≥ 30%	0.401	0.136	10	20%	8	16%	4	8
UW ≥ 25%	0.788	0.163	26	52%	12	24%	10	20%
**vHIT**	0.903	0.189						
Saccade			15	30%	3	6%	2	4%

FI: fixation instability; DP: directional preponderance; UW: unilateral weakness; The abbreviations for the remaining terms are explained as the same as Table 1. 0 week: baseline results; 2 weeks: results at 2 weeks after ST-PVR treatment; 4 weeks: results at 4 weeks after ST-PVR treatment

### Changes in the physical examination indexes results

**Fig. 2:**
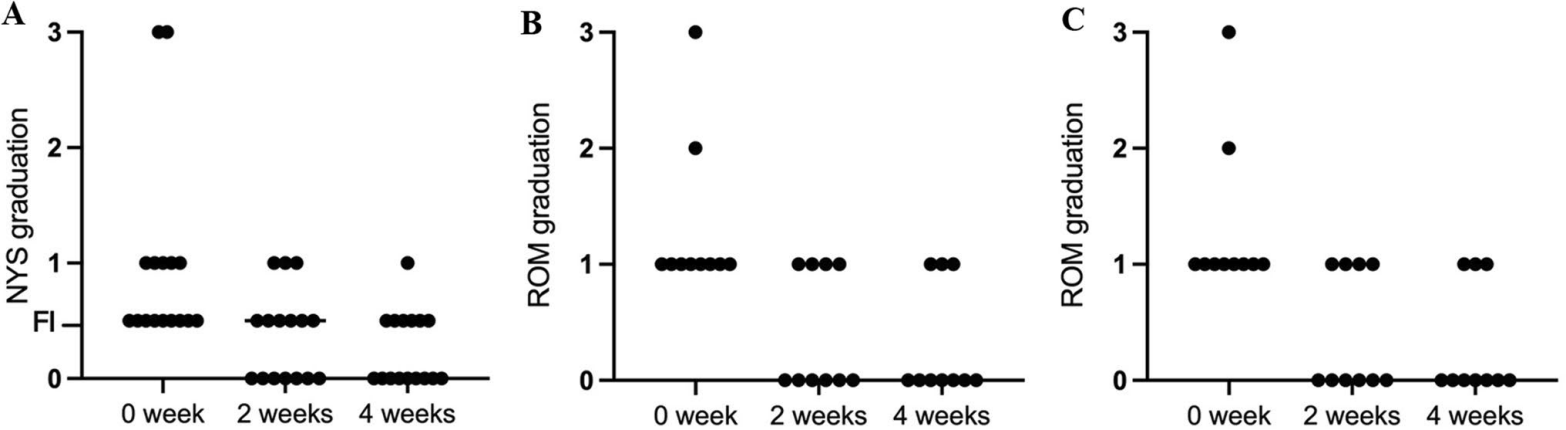
Changes in abnormal physical examination results of patients before and after treatment. **A** Distribution of patients at different grades of Nys (nystagmus) examination results from baseline to 2 and 4 weeks after treatment. **B** Distribution of patients at different graduation of Rom (Romberg test) examination results from baseline to 2 and 4 weeks after treatment. **C** Distribution of patients at different grades of HTT (head thrust test) examination results from baseline to 2 and 4 weeks after treatment. 0 week: baseline results; 2 weeks: results at 2 weeks after ST-PVR treatment; 4 weeks: results at 4 weeks after ST-PVR treatment

The proportions of patients with abnormal results in physical examination (Nys, Rom, and HTT) showed a decreasing trend over the course of ST-PVR treatment (Fig. 2), although no statistically significant differences were found. At baseline (0-week), the proportion of patients with abnormal eye movements was 32%; after 2 weeks of ST-PVR, it decreased to 20% and further decreased to 16% after 4 weeks. Initially, there were two patients with 3rd-grade nystagmus and five with 1st-degree nystagmus. However, after 4 weeks of ST-PVR, all nystagmus disappeared, except for one patient who had a slight residual 1st-grade nystagmus. The number of subjects without eye movement abnormalities who were completely normal increased from 0 at baseline to 9 after 4 weeks of intervention (Fig. 2A). In terms of ROM results, 10 patients had varying degrees of imbalance at baseline. After 4 weeks of ST-PVR, only three patients had mild imbalance, while the remaining seven patients returned to a normal balance status (Fig. 2B). The HTT results showed that three patients had saccades at baseline, but all returned to normal at 2 weeks after ST-PVR (Fig. 2C).

### Changes in the subjective scales results

#### VAS

**Fig. 3:**
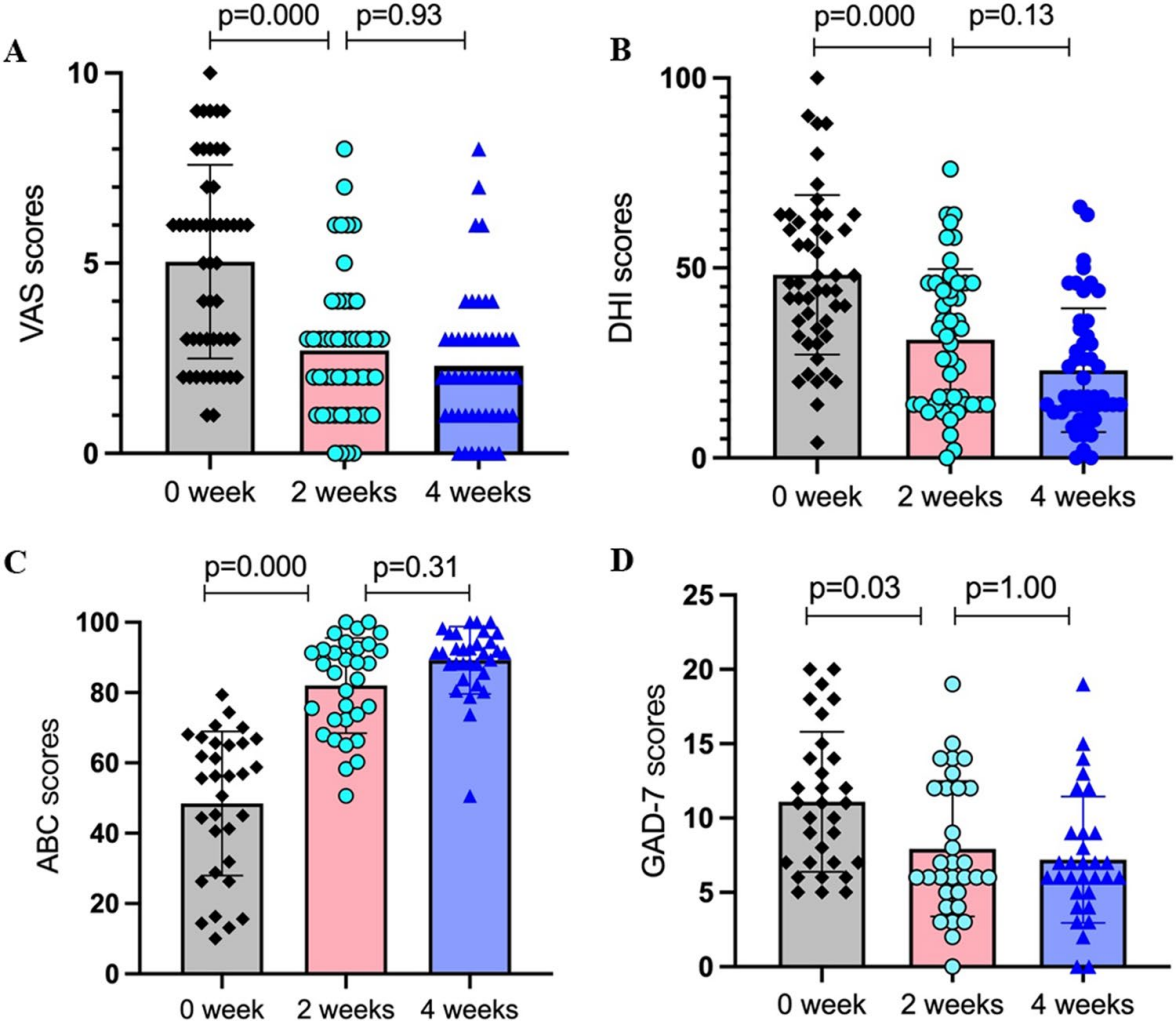
Changes in subjective scales results. **A** Changes in VAS scores at 0, 2, and 4 weeks after ST-PVR treatment. **B** Changes in DHI scores at 0, 2, and 4 weeks after ST-PVR treatment. **C** Changes in ABC scores at 0, 2, and 4 weeks after ST-PVR treatment. **D** Changes in GAD-7 scores at 0, 2, and 4 weeks after ST-PVR treatment. 0 week: baseline results; 2 weeks: results at 2 weeks after ST-PVR treatment; 4 weeks: results at 4 weeks after ST-PVR treatment; VAS: Visual Analogue scale; DHI: Dizziness Handicap Inventory scale; ABC: Activities-Specific Balance Confidence scale; GAD-7: Generalized Anxiety Disorder scale; Statistical significance: *P* < 0.05

The VAS scores (mean ± SD) at 0, 2, and 4 weeks after ST-PVR treatment were 5.04 ± 2.55, 2.70 ± 1.85 and 2.30 ± 1.80, respectively. Statistical analysis using the Kruskal‒Wallis *H* test indicated significant differences among the three groups (*H* = 33.96, *P* = 0.000). Pairwise comparison demonstrated a significant improvement in scores from 0 to 2 weeks after ST-PVR treatment (*P* = 0.000), while no significant difference was observed from 2 to 4 weeks after ST-PVR treatment (*P* = 0.93, Fig. 3A). Notably, two cases achieved a VAS score of 0 starting from the 2-week mark.

#### DHI

The DHI scores (mean ± SD) at 0, 2, and 4 weeks after ST-PVR treatment were 48.20 ± 21.00, 31.10 ± 18.59, and 23.08 ± 16.28, respectively. The Kruskal‒Wallis *H* test revealed significant differences among the three groups (*H* = 32.83, *P* = 0.000). Pairwise comparisons showed a significant improvement in scores from 0 to 2 weeks after ST-PVR treatment (*P* = 0.000), with no significant difference observed from 2 to 4 weeks after ST-PVR treatment (*P* = 0.13, Fig. 3B).

#### ABC

The ABC scores (mean ± SD) were 48.42 ± 20.50, 82.01 ± 13.55, and 89.21 ± 9.62, respectively. The Kruskal‒Wallis H test revealed significant differences among the ABC results at the three visits (*H* = 65.34, *P* = 0.000). Pairwise comparison showed a significant improvement in scores from 0 to 2 weeks after ST-PVR treatment (*P* = 0.000), while no significant difference was observed from 2 to 4 weeks after ST-PVR treatment (*P* = 0.31, Fig. 3C).

#### GAD-7

Out of the 50 patients, 31 patients with mild to moderate anxiety were assessed using the GAD-7 scale at baseline. The scores (mean ± SD) at 0, 2, and 4 weeks after ST-PVR treatment were 11.1 ± 4.71, 7.93 ± 4.56, and 7.19 ± 4.25, respectively. The Kruskal‒Wallis *H* test demonstrated significant differences among the GAD-7 results at the three visits (*H* = 39.62, *P* = 0.000). Pairwise comparisons showed a significant improvement in scores from 0 to 2 weeks after ST-PVR treatment (*P* = 0.03), while no significant difference was observed from 2 to 4 weeks after ST-PVR treatment (*P* = 1.00, Fig. 3D).

### Changes in the vestibular function test results

#### Using CDP to assess SOT

**Fig. 4:**
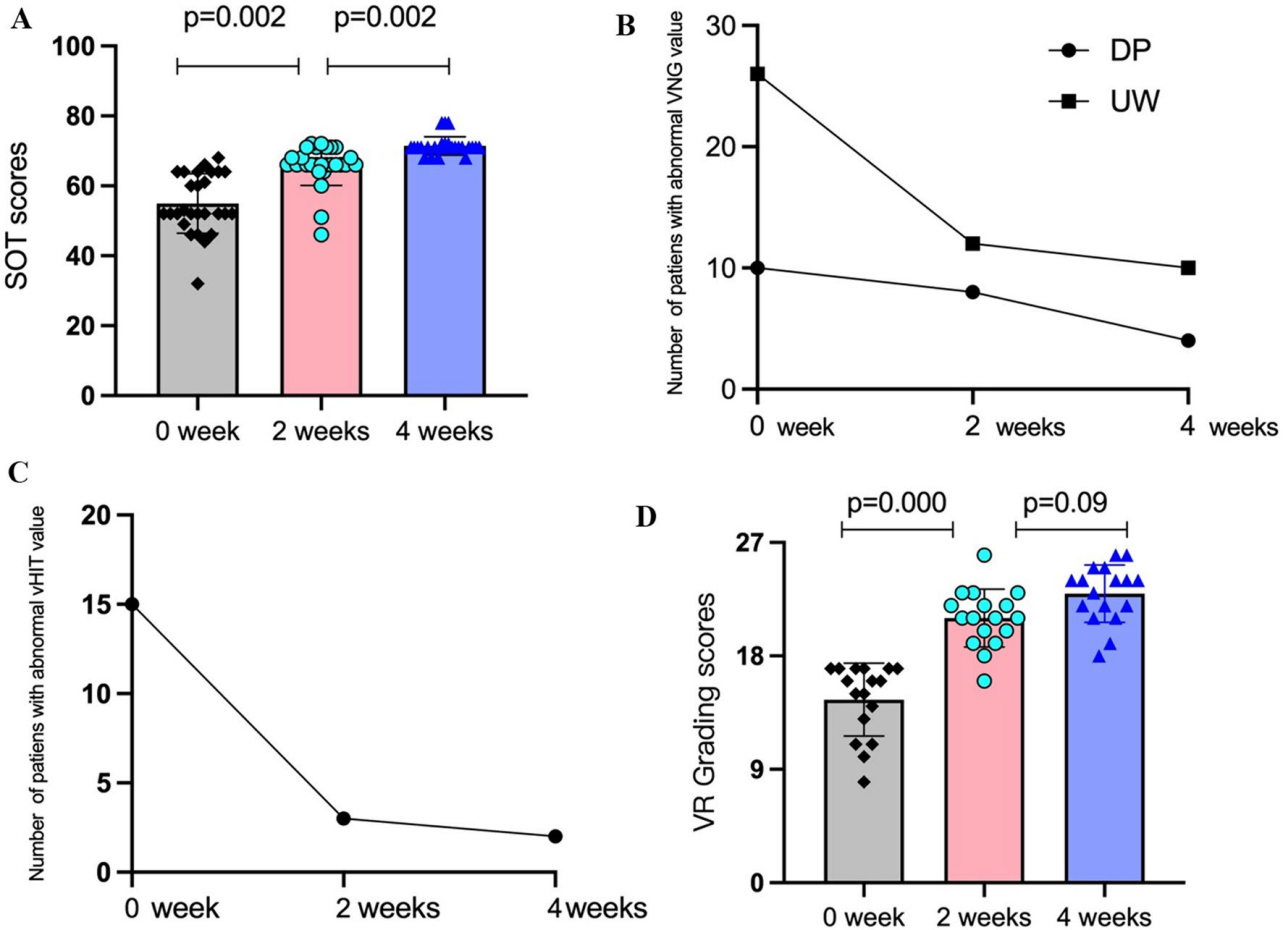
Changes in vestibular function tests results and vestibular rehabilitation efficacy grading scores. **A** Changes in SOT scores at 0, 2, and 4 weeks after ST-PVR treatment. **B** Changes in DP and UW scores in the VNG tests at 0, 2, and 4 weeks after ST-PVR treatment. **C** Changes in the abnormal rate of the vHIT at 0, 2, and 4 weeks after ST-PVR treatment. **D** Comparison of comprehensive grading scores for rehabilitation efficacy at 0-, 2-, and 4 week after ST-PVR treatment. 0 week: baseline results; 2 weeks: results at 2 weeks after ST-PVR treatment; 4 weeks: results at 4 weeks after ST-PVR treatment; DP: directional preponderance; UW: unilateral weakness; VNG: electronystagmography; vHIT: Video-Head Impulse Test; SOT: Sensory Organization Test; VR grading: Vestibular rehabilitation efficacy grading standard. Statistical significance: *P* < 0.05

During the initial evaluation, 27 patients (54%) exhibited abnormal SOT results. The mean ± SD scores at the 0-week, 2-week, and 4-week visits were 54.93 ± 8.50, 65.93 ± 5.81, and 71.44 ± 9.19, respectively. Significant improvements were observed in both the 0- to 2-week and 2- to 4-week periods following ST-PVR treatment (*P* = 0.002 for both). Only 4 cases demonstrated slightly abnormal scores at the 4-week mark after ST-PVR treatment (Fig. 4A).

#### CT using VNG test

Statistical analysis was conducted to analyze the number of patients with abnormal results during the three follow-up visits for both the VNG test and the vHIT test, as the results were presented in terms of the presence or absence of abnormalities rather than specific numerical values. The DP results in the VNG test were 20%, 16%, and 8% at the 0-, 2-, and 4-week visit time points, respectively. Although there was a trend of improvement in scores at 2 and 4 weeks after ST-PVR treatment, no statistically significant difference was observed (*χ *= 2.98, *P* = 0.23). The UW results were 52%, 24%, and 20% at the 0, 2, and 4 weeks visit time points, respectively. There was a significant decrease in abnormal rates at 2 weeks after ST-PVR treatment compared to the baseline data (*P* = 0.007), but no significant difference was found between the 2-week and 4-week after ST-PVR treatment (*P* = 0.81, Fig. 4B).

#### Vide-head impulse test (vHIT)

The abnormal rates of vHIT at the 0-, 2-, and 4-week visit time points were 30%, 6%, and 4%, respectively. There was a significant decrease in the abnormal rate at 2 weeks after ST-PVR treatment compared to the baseline data (*P* = 0.003). However, there was no significant difference between the 2-week and 4-week after ST-PVR treatment (*P* = 1.000, Fig. 4C).

Table 3 presents the integrated comparison and statistical analysis results of clinical indexes obtained from subjective scales, physical examination, and objective function evaluation at baseline, 2 week, and 4 week after ST-PVR treatment.

**Table 3:** Summary of the results from the comprehensive comparative and statistical analysis of all clinical indicators

Evaluation index	0 week	2 weeks	4 weeks	0–2	0–4	2–4
	Scores (mean ± SD)	Significantly difference				
Nys	–	–	–	ns	ns	Ns
Rom	–	–	–	ns	ns	Ns
HTT	–	–	–	ns	ns	Ns
VAS	5.04 ± 2.55	2.70 ± 1.85	2.30 ± 1.80	****	****	Ns
DHI	48.20 ± 21.00	31.10 ± 18.59	23.08 ± 16.28	****	****	Ns
ABC	48.42 ± 20.50	82.01 ± 13.55	89.21 ± 9.62	****	****	Ns
GAD-7	11.10 ± 4.71	7.93 ± 4.56	7.19 ± 4.25	*	**	Ns
SOT	54.93 ± 8.50	65.93 ± 5.81	71.44 ± 9.19	**	****	**
DP	–	–	–	ns	ns	Ns
UW	–	–	–	**	**	Ns
vHIT	–	–	–	**	**	Ns
VR Grading	14.52 ± 2.90	21.00 ± 2.29	22.94 ± 2.28	****	**	Ns

The abbreviations for the terms are explained as the same as the above tables and figures. 0–2: 0 week to 2 weeks after ST-PVR treatment. 0–4: 0 week to 4 weeks after ST-PVR treatment. 2–4: 2 weeks to 2 weeks after ST-PVR treatment. *p < 0.05; **p < 0.01; ***p < 0.001; ****p < 0.0001; ns: *p* ≥ 0.05

### Changes in the vestibular rehabilitation efficacy grading scores

According to the vestibular rehabilitation efficacy grading standard provided by the Chinese Experts Consensus in Vestibular Rehabilitation (Table 1), nine clinical indicators were used to assess different clinical performances. The total grading scores were calculated at three visits during the study: baseline, 2 weeks, and 4 weeks after ST-PVR treatment. The results demonstrated that the scores (mean ± SD) at baseline, 2 weeks, and 4 weeks after ST-PVR treatment were 14.52 ± 2.90, 21.00 ± 2.29, and 22.94 ± 2.28, respectively. A statistically significant improvement was observed in the score in the 2 weeks after ST-PVR treatment compared to baseline (*P* = 0.000). However, no significant difference was found between the scores at the 2-week and 4-week after ST-PVR treatment, although there was a noticeable trend of improvement (*P* = 0.09). (Fig. 4D).

## Discussion

In benign recurrent vestibular vertigo, patients commonly experience episodes of dizziness and unsteadiness, which occur frequently but do not present with other neurological symptoms. However, it is noteworthy that many patients also develop psychological disorders, including anxiety, due to the impact of their condition. While pharmacological interventions are often insufficient in providing optimal treatment outcomes, vestibular rehabilitation training has emerged as an effective therapeutic approach. This specialized training aims to expedite the establishment of stable compensation within the central vestibular system [20, 38]. By enhancing the brain's ability to adapt and compensate for vestibular dysfunction, vestibular rehabilitation plays a crucial role in controlling the recurrent nature of the condition. Vestibular rehabilitation training encompasses a variety of exercises and techniques tailored to the individual's specific needs. These exercises typically involve head and body movements, balance training, and visual stabilization exercises. The overall objective is to recalibrate the vestibular system, improve balance, and enhance overall functional abilities. Numerous studies have demonstrated the effectiveness of vestibular rehabilitation in reducing the frequency and severity of recurrent vertigo episodes [39]. Patients who undergo vestibular rehabilitation often report a significant improvement in their symptoms, leading to enhanced quality of life and reduced psychological distress. It is important to note that vestibular rehabilitation should be customized based on individual patient characteristics, such as the type and severity of vestibular dysfunction. A multidisciplinary approach involving healthcare professionals specialized in vestibular rehabilitation can provide personalized treatment plans and ensure the best possible outcomes for patients.

Vestibular compensation is a complex process that involves both static and dynamic mechanisms. Static compensation refers to the restoration of balance and reduction in dizziness symptoms that occur when the patient is in a stationary position. On the other hand, dynamic compensation relates to the improvement in balance and reduction in dizziness symptoms during active movements and more challenging daily activities. The establishment of static compensation is indicated by the disappearance of subjective symptoms, such as dizziness or vertigo, as reported by the patient. Objective signs, such as abnormal eye movements or postural instability, also diminish or resolve. This suggests that the central nervous system has adapted to vestibular dysfunction, allowing the patient to maintain stability during static conditions. Dynamic compensation, on the other hand, involves adaptation of the central nervous system to maintain balance and stability during dynamic activities. For example, patients may experience a reduction in dizziness and unsteadiness while walking, running, or performing tasks that require head and body movements. The ability to maintain balance during such activities indicates the establishment of dynamic compensation.

To assess the compensation process and determine its establishment, various subjective and objective measures are utilized. Commonly employed subjective measures include validated questionnaires that assess the severity and frequency of dizziness symptoms, as well as the impact on daily functioning and quality of life. Physical examinations, including assessments of eye movements, balance, and postural control, can provide objective insights into the patient's compensatory abilities. In addition to subjective questionnaires and physical examinations, specific objective evaluation tests may be employed. These tests can include VNG and vHIT, which can assess eye movements under external stimulus conditions and during head movements and SOT, which evaluates postural control under various conditions. These assessments provide quantitative data that can help monitor the progress of compensation and determine the effectiveness of treatment interventions. The use of these assessment tools and measures allows healthcare professionals to track the progress of vestibular compensation and tailor treatment approaches accordingly. By evaluating the clinical indicators mentioned earlier, clinicians can gain a comprehensive understanding of the patient's compensatory status and make informed decisions regarding the management of benign recurrent vestibular vertigo.

ST-PVR incorporates three key features that contribute to its effectiveness in treating decompensated vestibular vertigo patients. First, it adopts a short duration rehabilitation program in everyday training. This time-limited approach is designed to achieve efficient and rapid improvements in vestibular function and compensation by providing focused interventions within a condensed timeframe.ST-PVR aims to minimize the training burden while achieving an optimal rehabilitation process. Second, ST-PVR emphasizes a personalized approach to rehabilitation. The program is customized to each patient's specific condition, taking into account factors such as the underlying vestibular pathology, functional deficits, and individual goals. This individualized approach ensures that the rehabilitation interventions are tailored to address the unique needs of each patient. By targeting specific areas of impairment and considering individual factors, ST-PVR optimizes the effectiveness of the treatment and improves outcomes. Lastly, ST-PVR involves intensive rehabilitation sessions. During these sessions, patients receive frequent and focused interventions over a condensed period. The intensity of treatment aims to maximize the potential for vestibular compensation and functional improvement within a shorter timeframe. By providing concentrated and targeted interventions, ST-PVR promotes efficient adaptation and enhances the potential for a successful rehabilitation outcome.

ST-PVR demonstrates significant advantages in the treatment of uncompensated vestibular vertigo [17]. This study also showed similar results in patients with uncompensated recurrent vertigo. In subjective assessment scales, including VAS, DHI, ABC, and GAD-7, statistically significant differences were observed after 2 weeks of treatment, indicating that ST-PVR has an ideal therapeutic effect in improving the severity of dizziness, the impact of the disease on functional abilities, self-confidence in maintaining balance, and reducing anxiety levels. Among these scales, VAS, ABC, and GAD-7 scores reached normal or near-normal levels after 2 weeks of treatment, resulting in no significant differences between the 2- and 4-week results. Only the DHI scale showed continued improvement after 4 weeks of treatment compared to the 2-week results, suggesting a slightly slower rate of improvement in the comprehensive DHI index compared to other scale indicators. Physical examinations in this study included Nys, HTT, and ROM, representing static compensation status, dynamic compensation of the VOR pathway function, and balance (including VSR pathway) function, respectively. These assessment methods were not precise enough, resulting in a smaller number of individuals with clearly positive signs. Therefore, no statistically significant differences were observed in the results after treatment. However, a gradual improvement process was still evident as the treatment progressed. Objective evaluation measures included commonly used clinical tests such as UW and DP in VNG, SOT and vHIT. The results reflecting VOR pathway function, indicated by CT and vHIT, showed statistically significant improvements after 2 weeks of treatment. However, improvements in balance (including the VSR pathway) function, assessed by the SOT, were observed after 4 weeks of treatment, suggesting that the vestibular compensation rate of the VOR pathway may be superior to that of the VSR pathway.

The mentioned indicators effectively demonstrate the process of vestibular compensation and indicate the establishment of static and dynamic compensation. Integrating various indicators that reflect compensation changes for a comprehensive assessment will provide a more comprehensive reflection of the compensation status. According to the Vestibular Rehabilitation Efficacy Assessment Criteria proposed by Chinese vestibular rehabilitation experts, each indicator is scored based on the degree of improvement, and the comprehensive scores before and after treatment are calculated and compared. The results show statistically significant improvement after 2 weeks of treatment. Therefore, the graded scoring of the VR comprehensive score is also an effective indicator that can comprehensively reflect the vestibular compensation status and effectively monitor the results of vestibular compensation.

Our next step involves the continuation of patient observation and follow-up, employing a long-term monitoring approach to investigate the relapse of BRV symptoms and assess the effectiveness of ST-PVR in their control, while also assessing the potential instances of treatment failure. Thorough and comprehensive follow-up assessments will provide us with deeper insights into the long-time dynamics of BRV. Additionally, we are considering expanding the scope of the current study by examining various variables in the included patients, such as different personality traits, psychological characteristics, and underlying medical conditions, as these factors may impact treatment outcomes. We will increase our sample size and conduct comparative studies, aiming to identify the direct factors that influence treatment outcomes. Furthermore, we plan to conduct additional randomized controlled studies to compare the differential advantages and disadvantages of ST-PVR treatment with other therapeutic approaches in facilitating vestibular compensation. These studies aim to provide more evidence-based medical evidence to optimize treatment strategies.

## Conclusion

The research findings indicate that short-duration personalized vestibular rehabilitation training has a clear and effective therapeutic effect on patients with uncompensated benign recurrent vertigo. The severity of dizziness, balance function, daily life abilities, and psychological state of the patients showed significant improvement after 2 weeks of treatment. By integrating subjective scales, physical examinations, and objective evaluations, the graded scoring of the VR comprehensive score can comprehensively reflect the changes in the vestibular compensation status of patients before and after treatment. Therefore, it is worth promoting as a comprehensive assessment approach for evaluating the rehabilitation of vestibular function.
